# Inhibitory Mechanism of Bee Venom Against A431 Cutaneous Squamous Cell Carcinoma Cells Based on Label-Free Proteomics and Untargeted Metabolomics

**DOI:** 10.3390/ijms27198725

**Published:** 2026-09-29

**Authors:** Ao Yang, Jingjing Li, Wenchao Yang

**Affiliations:** College of Bee Science, Fujian Agriculture and Forestry University, Fuzhou 350002, China; ya104110@126.com (A.Y.); lijingjing000407@163.com (J.L.)

**Keywords:** Bee venom (BV), A431 cells, label-free proteomics, untargeted metabolomics

## Abstract

The incidence of cutaneous squamous cell carcinoma (cSCC) has been rising steadily in recent years, yet treatment options for advanced-stage patients remain limited. Bee venom (BV) possesses diverse biological activities including inhibiting tumor cell growth in various tumor models, but its underlying mechanism against cSCC A431 cells remains unclear. This study evaluated the antiproliferative effect of BV on A431 cells using the CCK-8 assay and explored its mechanism of action through label-free proteomics and untargeted metabolomics. BV inhibited A431 cell proliferation in a dose-dependent manner, with a half-maximal inhibitory concentration of 11.57 μg/mL. Proteomics analysis revealed that 118 differentially expressed proteins were mainly enriched in the p53 signaling pathway, mineral absorption, and base excision repair pathways. Metabolomics screening yielded 336 differentially expressed metabolites, primarily enriched in amino acid biosynthesis and metabolism. BV exerts its anti-A431 cell bioactivity potentially by synergistically regulating tumor-related signaling pathways, mineral homeostasis, and proteins involved in DNA repair processes, thereby disrupting amino acid biosynthesis and metabolism. But the transcriptomic data failed to confirm *TP53* upregulation. These findings provide a theoretical basis for apipuncture and BV-based cSCC therapies.

## 1. Introduction

Bee products, as a traditional natural resource, have long been widely used in food nutrition and human health [1]. Bee products have diverse chemical compositions, containing a variety of components such as carbohydrates, proteins, polypeptides, enzymes, and phenolic compounds, and exhibit biological activities such as antioxidant, antibacterial, anti-inflammatory, immunomodulatory and cytoprotective effects. Therefore, bee products have received increasing attention as a source of highly promising functional food ingredients and natural bioactive additives [2]. However, the composition and bioactivity of bee products vary depending on the plant source, geographical origin, bee species, collection conditions and processing methods [3]. Therefore, systematic characterization of their bioactive properties and potential molecular mechanisms is crucial for their rational utilization in food-related applications [1].

Bee venom (BV) is a pale yellow, transparent secretion from the venom glands of worker bees and is a complex mixture containing a variety of bioactive components including peptides, enzymes, and small molecule compounds [4]. Melittin is the main active component, accounting for about 40% to 50% of the dry weight of BV. It has an amphiphilic α-helical structure and disrupts cell membrane integrity and produces strong cytotoxicity [5,6]. Phospholipase A_2_ accounts for 10% to 12% of the dry weight of BV and participates in cell membrane remodeling and toxin activation processes [7,8]. Other key components include apamin, MCD peptides, hyaluronidase, histamine, dopamine, etc. [9]. Numerous studies have confirmed that BV has a variety of biological activities, including anti-inflammatory [10], antibacterial [11], antiviral [12], antitumor [13], radiation protection [14], and analgesic [15]. Among them, the antitumor potential of BV has received widespread attention. Existing studies have confirmed that BV and its active ingredients can inhibit the proliferation, migration and invasion of various tumor cells by inducing apoptosis, regulating immune responses and disrupting cell membrane structure [5,16].

Cutaneous squamous cell carcinoma (cSCC) is the second most common non-melanoma skin cancer worldwide, accounting for approximately 20% of all skin malignancies [17]. In recent years, the incidence of cSCC has been increasing year by year, especially in the elderly population, and the incidence in men is significantly higher than that in women, with a male-to-female ratio of about 3:1 [18]. Most cSCC cases develop gradually from precancerous lesions, among which actinic keratosis is the most common precancerous lesion and the earliest clinically identifiable precursor lesion, mainly manifested as scaly papules or plaques on an erythematous base [19]. The occurrence of cSCC is affected by a variety of factors, including long-term ultraviolet radiation, HPV infection, persistent immunosuppression after transplantation, chronic skin diseases, genetic diseases, ionizing radiation, and chronic arsenic exposure [20]. From a molecular perspective, the malignant transformation and progression of cSCC mainly involve multiple aspects, including key driver gene mutations, tumor cell metabolic reprogramming, and epigenetic regulation. Currently, 23 mutated genes closely related to cSCC have been identified, such as *TP53*, *CDKN2A*, *NOTCH1*, *NOTCH2*, and *RAS*. Among them, *TP53* has a mutation frequency of 54–95% in cSCC patients and is a key driver in the early stages of tumor development [21]. Metabolic reprogramming is an important characteristic of cSCC and other malignant tumors, with the Warburg effect being the most representative. Even under normoxic conditions, tumor cells preferentially use glycolysis for energy to support their rapid growth and biosynthetic needs [22,23]. Epigenetic alterations, including DNA methylation, histone modification, and miRNA dysregulation, can regulate gene expression without altering the DNA sequence, playing a crucial role in the malignant transformation and progression of cSCC [24,25].

Currently, the mainstream clinical treatment for cSCC is surgical resection and chemotherapy, supplemented by curettage, cryotherapy, photodynamic therapy and laser therapy [26]. Although surgical resection has a high cure rate for early cSCC, 2% to 6% of patients still experience local recurrence and metastasis after surgery. Surgical intervention is not suitable for patients with advanced and metastatic cSCC, and they have low sensitivity to chemotherapy drugs, so their overall prognosis is poor [27,28]. Therefore, it is of great research significance to find natural active substances with antitumor activity to provide new strategies for the treatment of cSCC.

To elucidate the antitumor effects and underlying molecular mechanisms of BV on cSCC A431 cells, the effect of BV on A431 cell proliferation and its half-maximal inhibitory concentration (IC_50_) were detected using the CCK-8 assay. Subsequently, label-free proteomics and untargeted metabolomics analyses were combined to screen differentially expressed proteins (DEPs) and metabolites (DEMs) in A431 cells treated with BV to elucidate the involved biological processes and related signaling pathways. Collectively, these findings provide experimental evidence and a theoretical reference for BV’s antitumor activity against A431 cells for its development and application as bee-derived natural antitumor drugs.

## 2. Results

### 2.1. The Morphology and Antitumor Effect of BV Against A431

**Figure 1:**
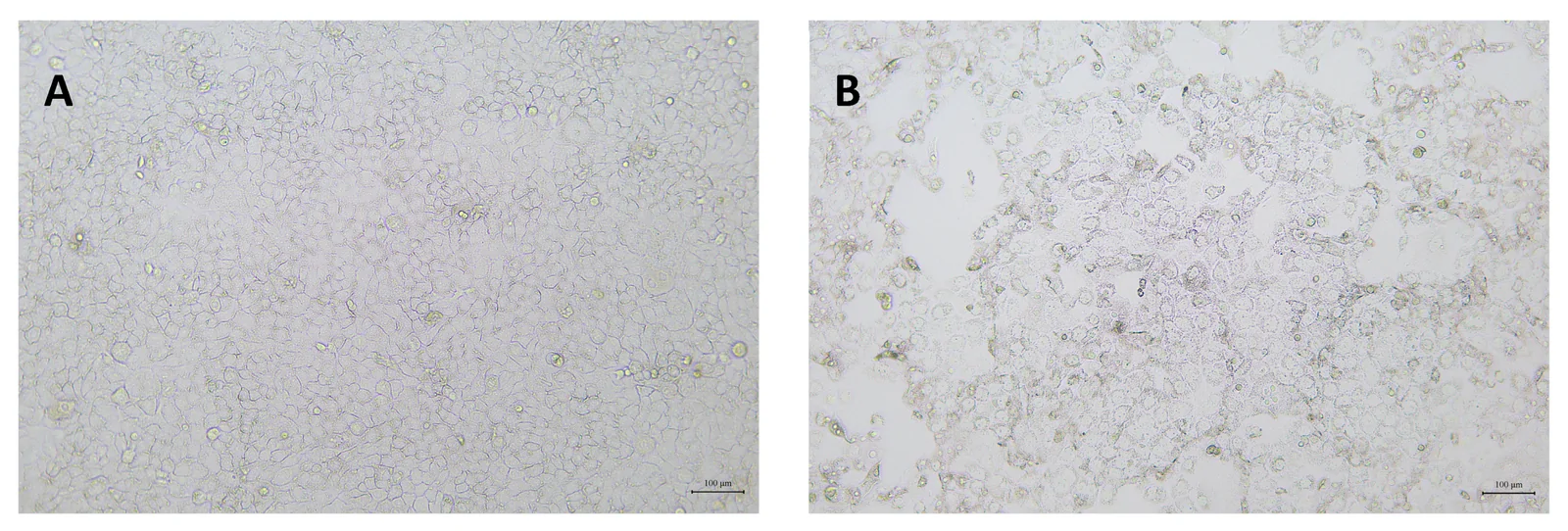
Effect of BV on the morphology of A431 cells (100×). (**A**) Blank control group; (**B**) BV treatment group.

The concentration of melittin in BV is 59.84 ± 0.01%. The morphology of A431 is shown in Figure 1. The cells in the control group had regular morphology, grew in sheets, and are in good condition. After 24 h of BV treatment, the number of cells decreased significantly and the cell volume shrank, suggesting that BV can inhibit the growth of A431 cells and cause changes in cell morphology.

**Figure 2:**
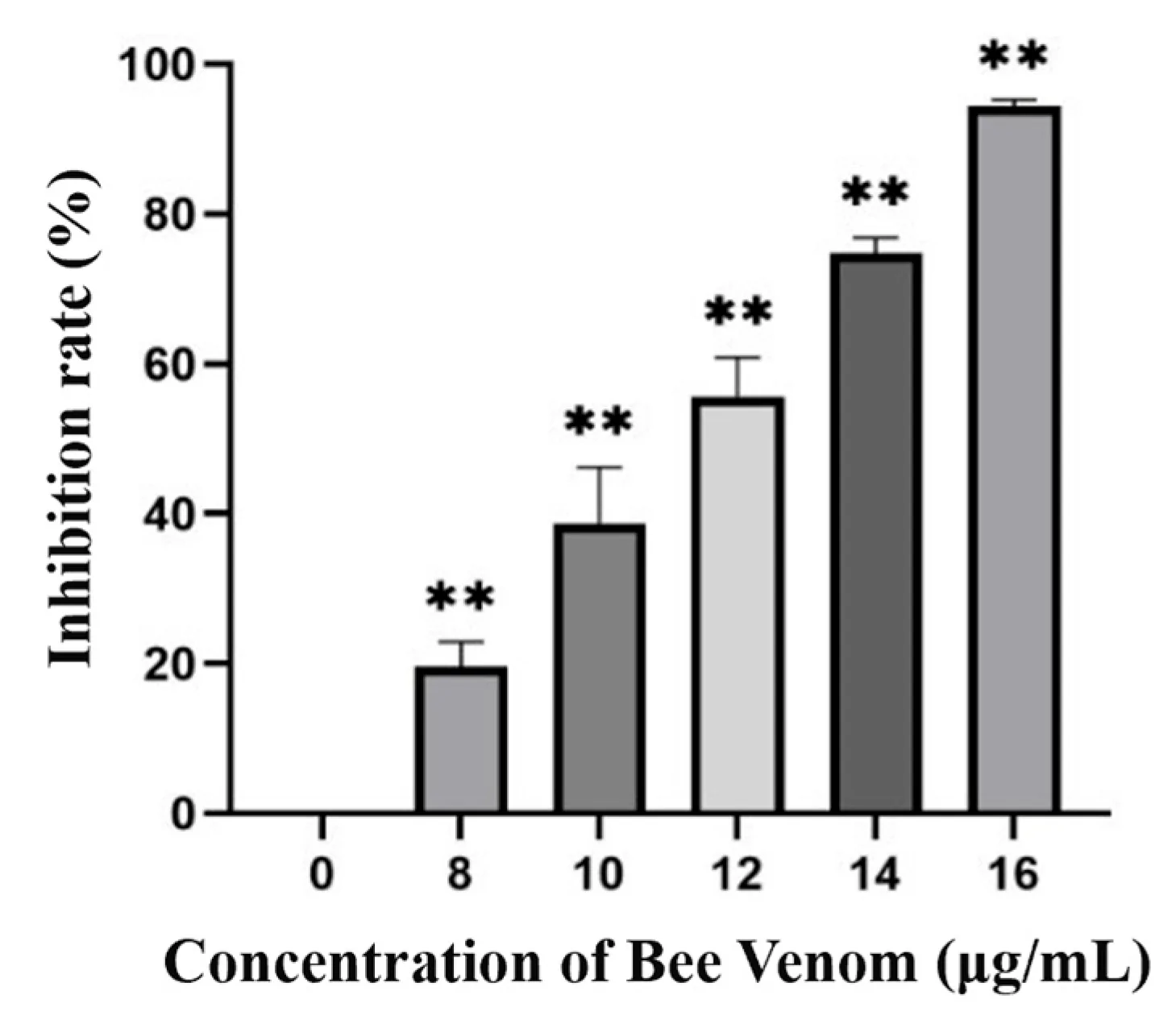
Inhibitory effects of BV on the proliferation of A431 cells. Data are presented as the mean ± SE of three independent biological experiments, with 6 technical replicates per experiment (*n* = 3). ** Significant differences compared with the blank control group (*p* < 0.01).

The CCK-8 assay results are shown in Figure 2. With increasing BV concentration, the proliferation of A431 cells is significantly inhibited in a dose-dependent manner. After 24 h of BV treatment, the IC_50_ for A431 cells is 11.57 μg/mL, indicating that BV has a strong inhibitory activity against the proliferation of A431 cells.

### 2.2. The Differentially Expressed Proteins

**Figure 3:**
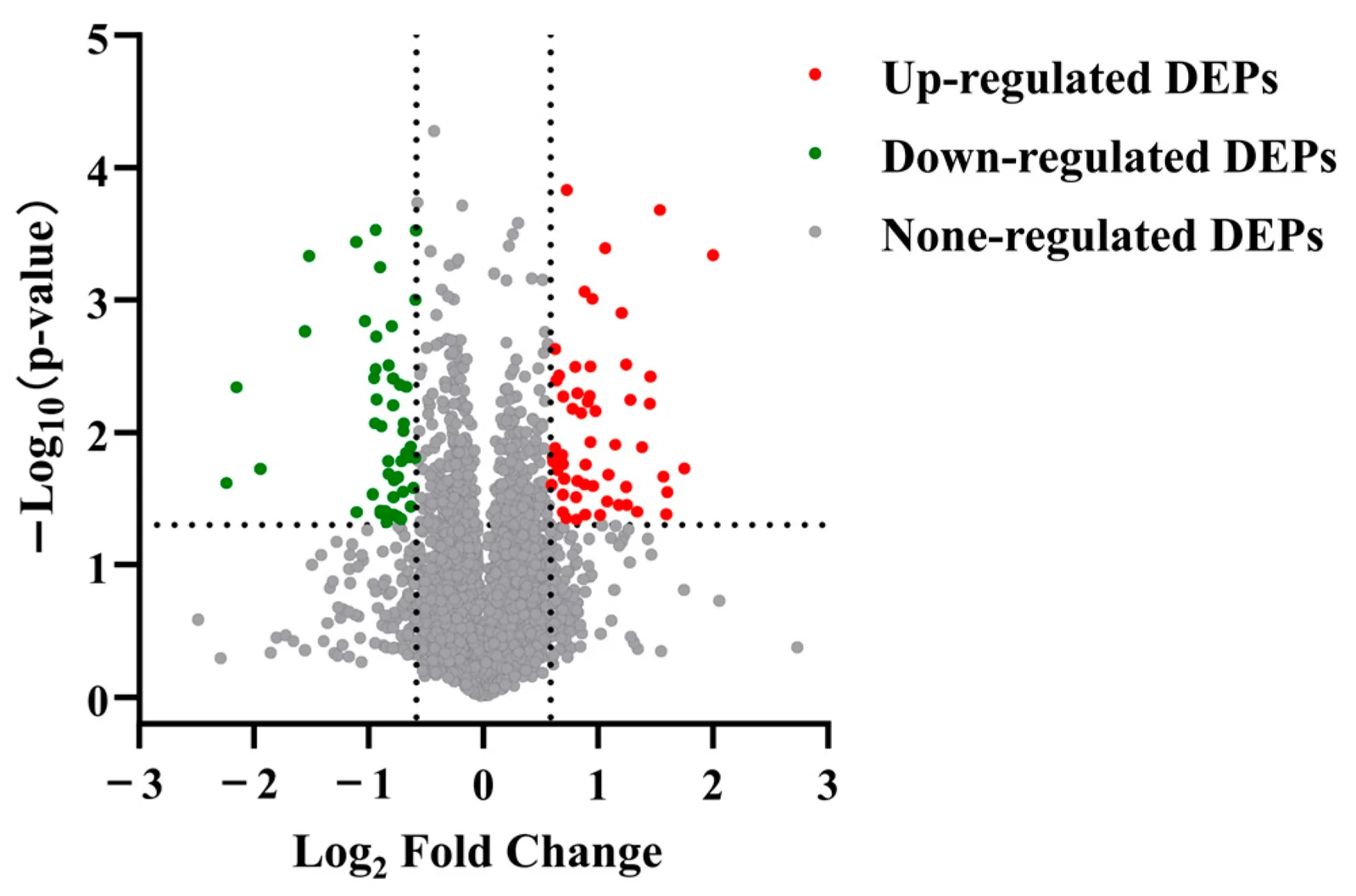
Volcano plot of proteins in A431 cells between the BV treatment group and the blank control group.

Comparative proteomic analysis is performed between the BV group and the control group using FC > 1.5 or FC < 0.67 and *p* < 0.05 as the screening criteria of DEPs. There are 61 upregulated and 57 downregulated DEPs. The top 15 up- or downregulated DEPs are listed in Table 1. The volcano plot of the two groups of differential proteins is shown in Figure 3.

**Figure 4:**
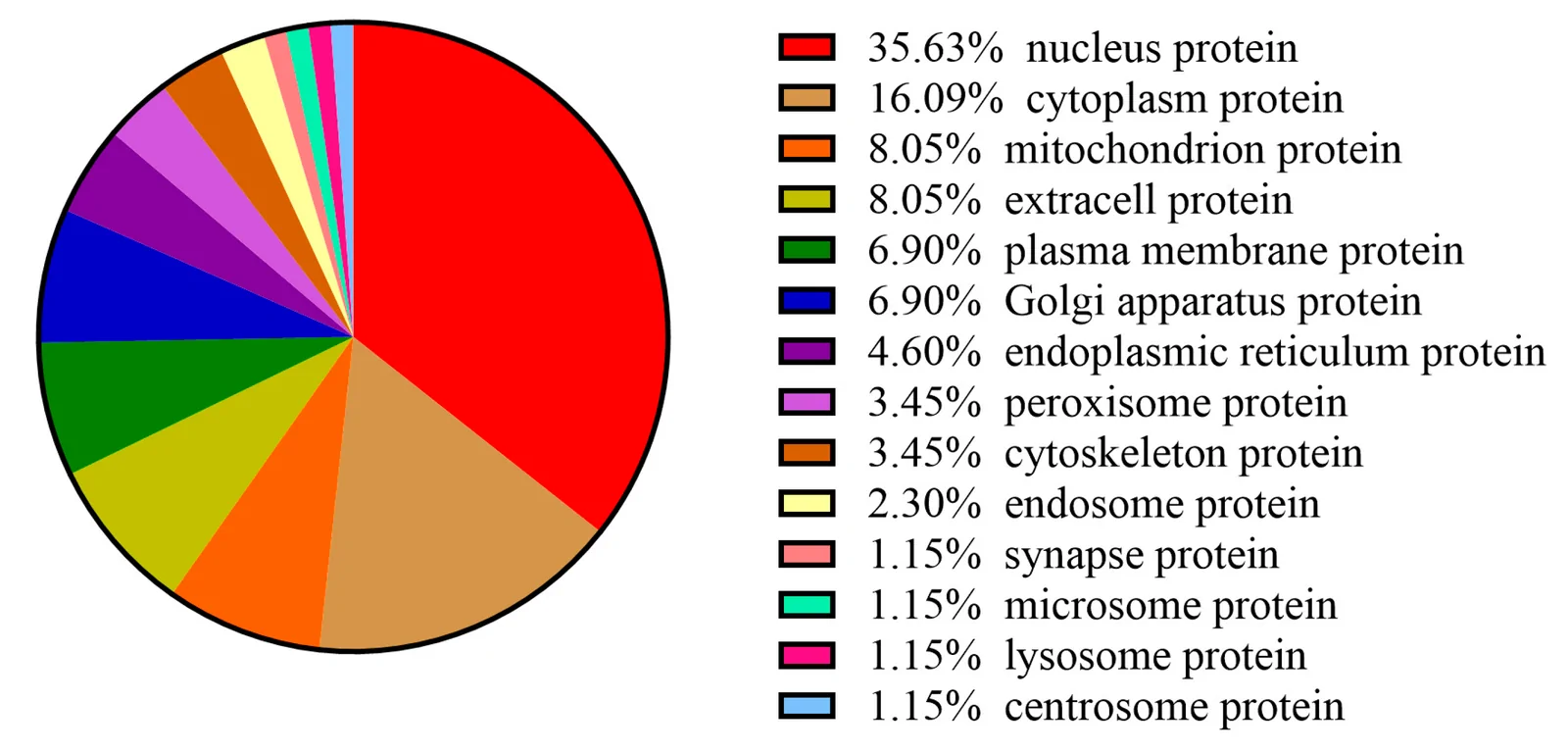
The subcellular localization of 87 annotated DEPs out of 118 total DEPs in A431 cells following BV treatment (A slight 0.02% discrepancy is due to rounding to two decimal places.).

Among the 118 identified DEPs (61 upregulated, 57 downregulated), 87 DEPs possess confirmed subcellular localization annotations and are subsequently categorized in Figure 4. They are mainly located in the nucleus (35.63%), followed by the cytoplasm (16.09%). Furthermore, mitochondria and extracellular regions each accounted for 8.05%, the plasma membrane and Golgi apparatus each accounted for 6.90%, the endoplasmic reticulum accounted for 4.60%, peroxisomes and the cytoskeleton each accounted for 3.45%, endosomes accounted for 2.30%, and synapses, microsomes, lysosomes, and centrosomes each accounted for 1.15%.

**Figure 5:**
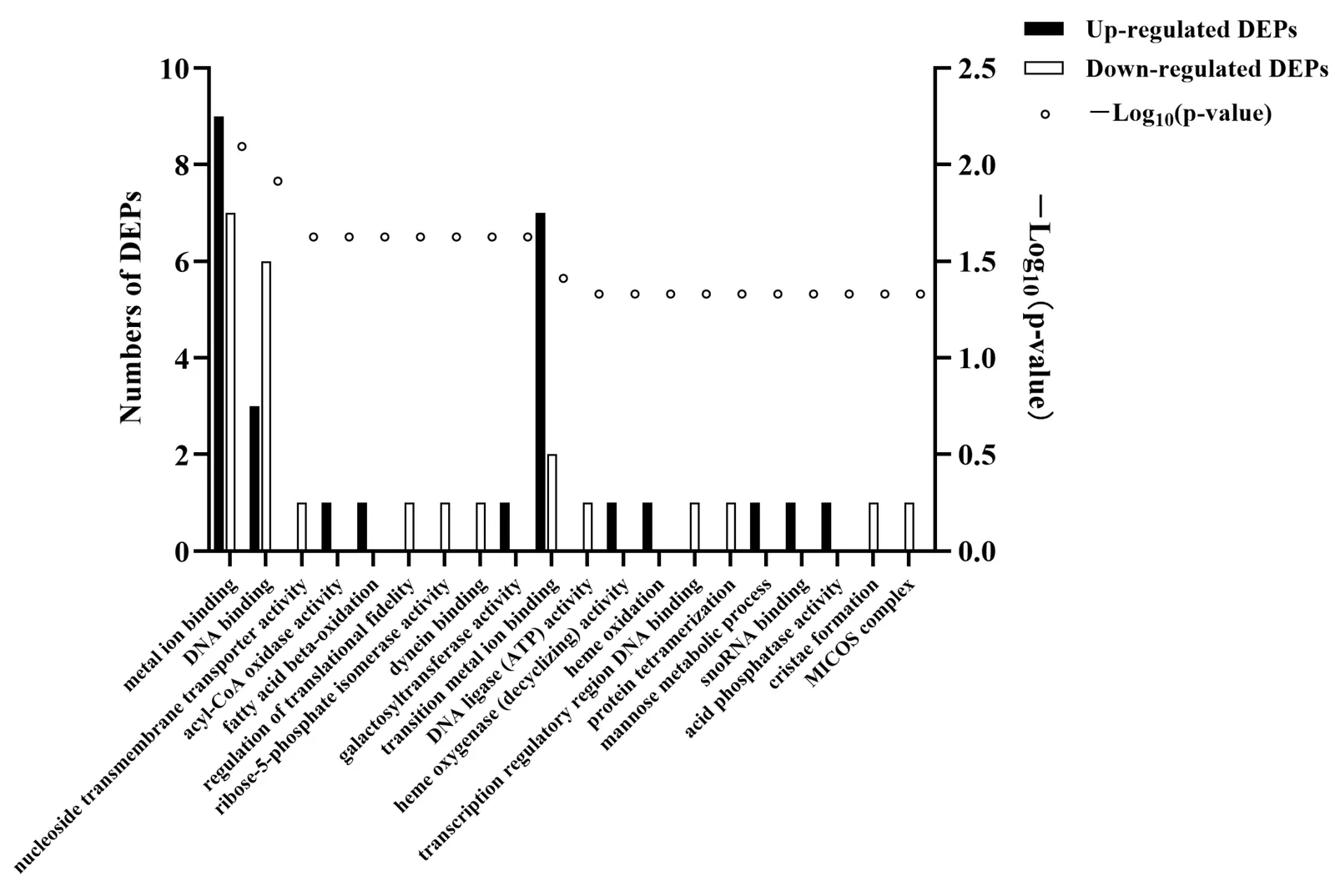
The significantly enriched Gene Ontology terms (*p* < 0.05).

The results of GO enrichment analysis are shown in Figure 5. A total of 92 GO items were enriched, of which 20 reached a significant level (*p* < 0.05). In biological processes (BP), DEPs are mainly enriched in fatty acid beta-oxidation, regulation of translational fidelity, heme oxidation, protein tetramerization, mannose metabolic process, and cristae formation items. In cellular components (CC), only the MICOS complex item was significantly enriched. In molecular functions (MF), a total of 13 items are significantly enriched, including metal ion binding, DNA binding, nucleoside transmembrane transporter activity, acyl-CoA oxidase activity, ribose-5-phosphate isomerase activity, dynein binding, galactosyltransferase activity, transition metal ion binding, DNA ligase (ATP) activity, heme oxygenase (decyclizing) activity, transcription regulatory region DNA binding, snoRNA binding, and acid phosphatase activity items.

**Figure 6:**
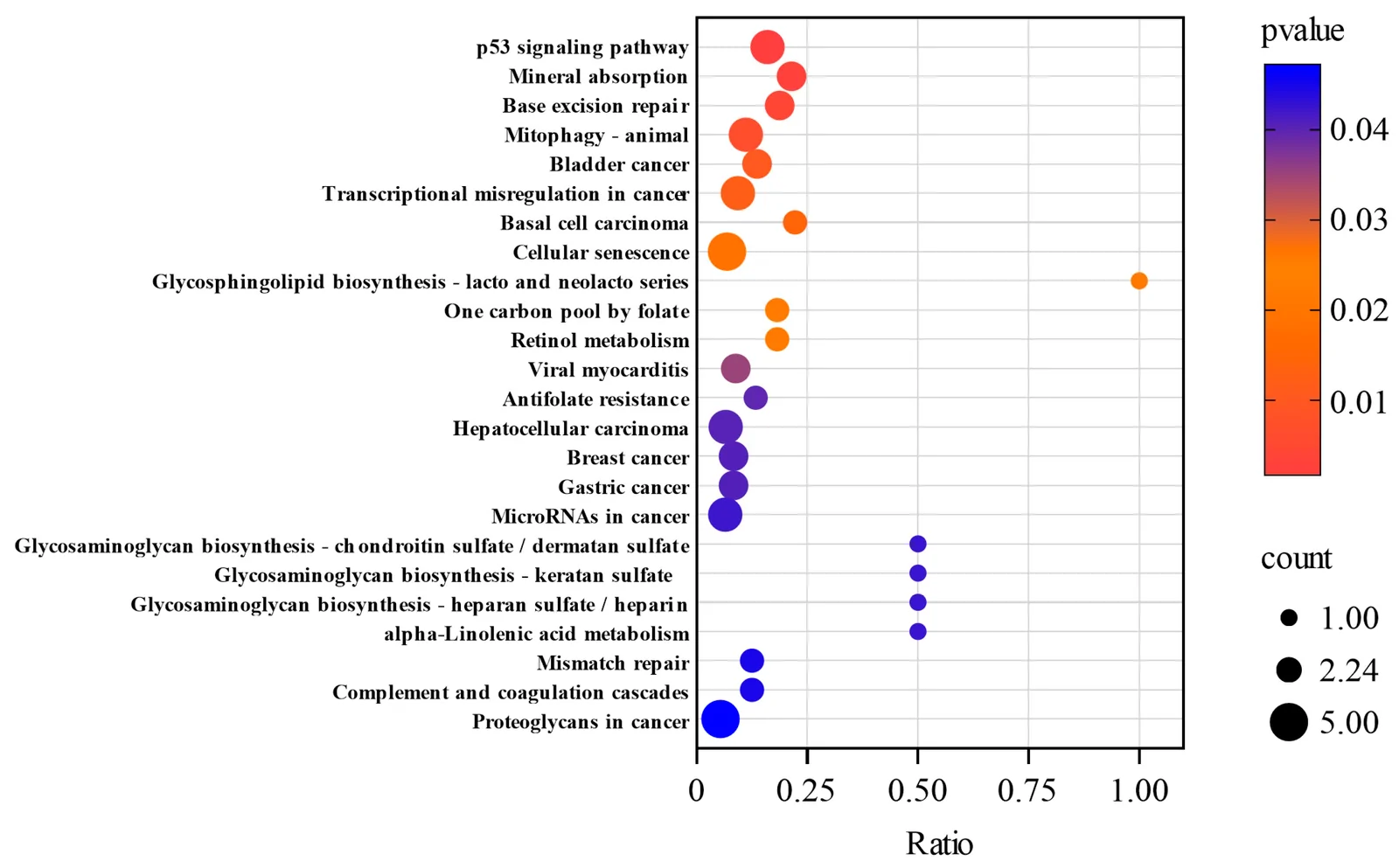
The significantly enriched pathways of DEPs (*p* < 0.05).

The results of the KEGG pathway enrichment analysis are shown in Figure 6. A total of 118 signaling pathways are enriched, of which 24 are significant (*p* < 0.05). Detailed information is provided in Supplementary Table S1. The significantly enriched pathways mainly included the p53 signaling pathway, mineral absorption, base excision repair, et al.

### 2.3. The Expression of the Hub Genes

**Figure 7:**
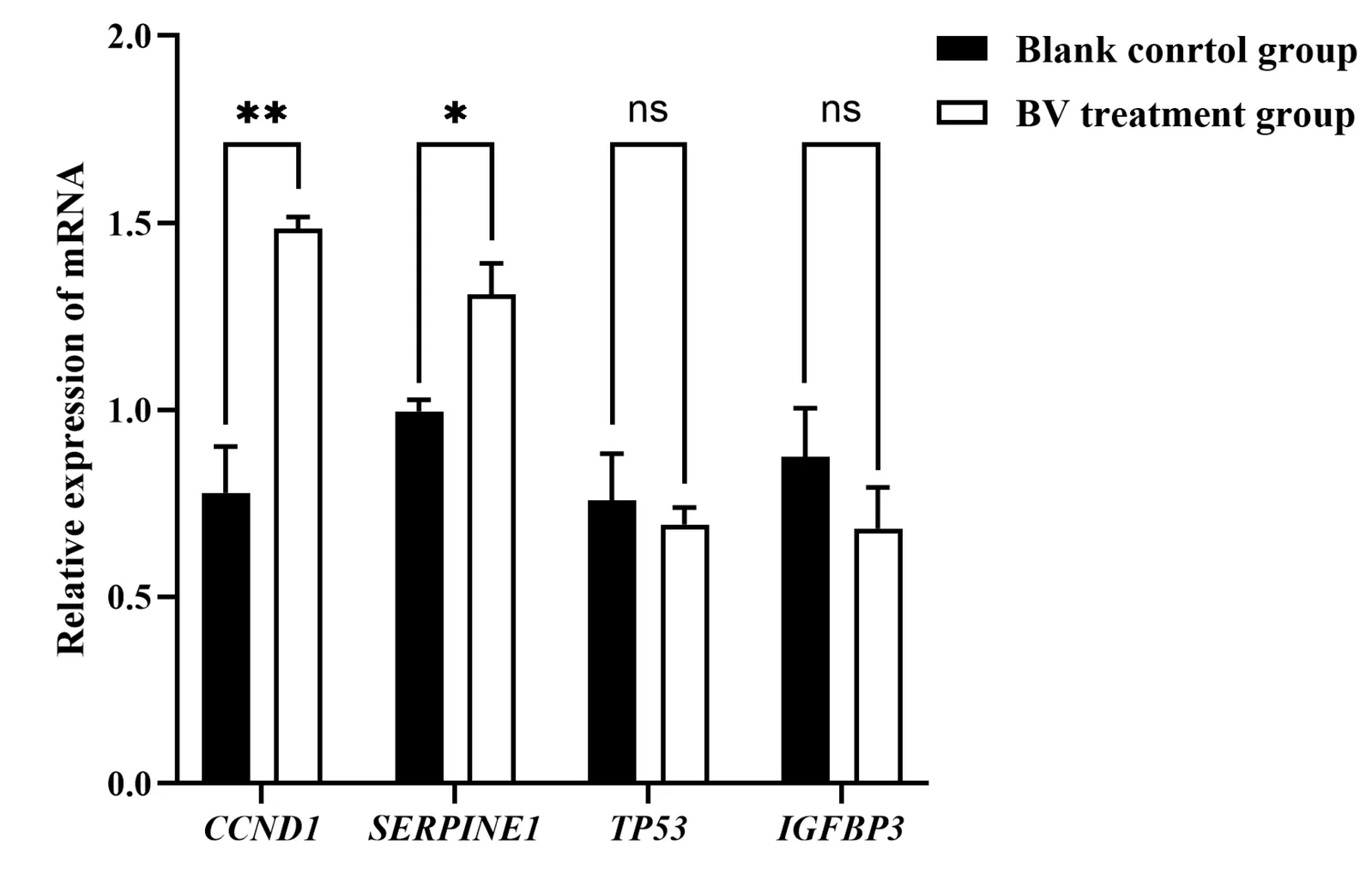
qRT-PCR validation of *CCND1*, *SERPINE1*, *TP53*, and *IGFBP3* expression in A431 cells after BV treatment. Compared with the blank control group, ns represents *p* > 0.05, * represents *p* < 0.05, and ** represents *p* < 0.01.

The qRT-PCR results are shown in Figure 7. Compared with the blank control group, the mRNA expression levels of CCND1 and SERPINE1 in A431 cells were significantly increased after BV treatment. The direction of changes for both at the transcriptional level was consistent with the proteomics results, further supporting the expression change trends detected by label-free proteomics.

### 2.4. The Differentially Expressed Metabolites

**Figure 8:**
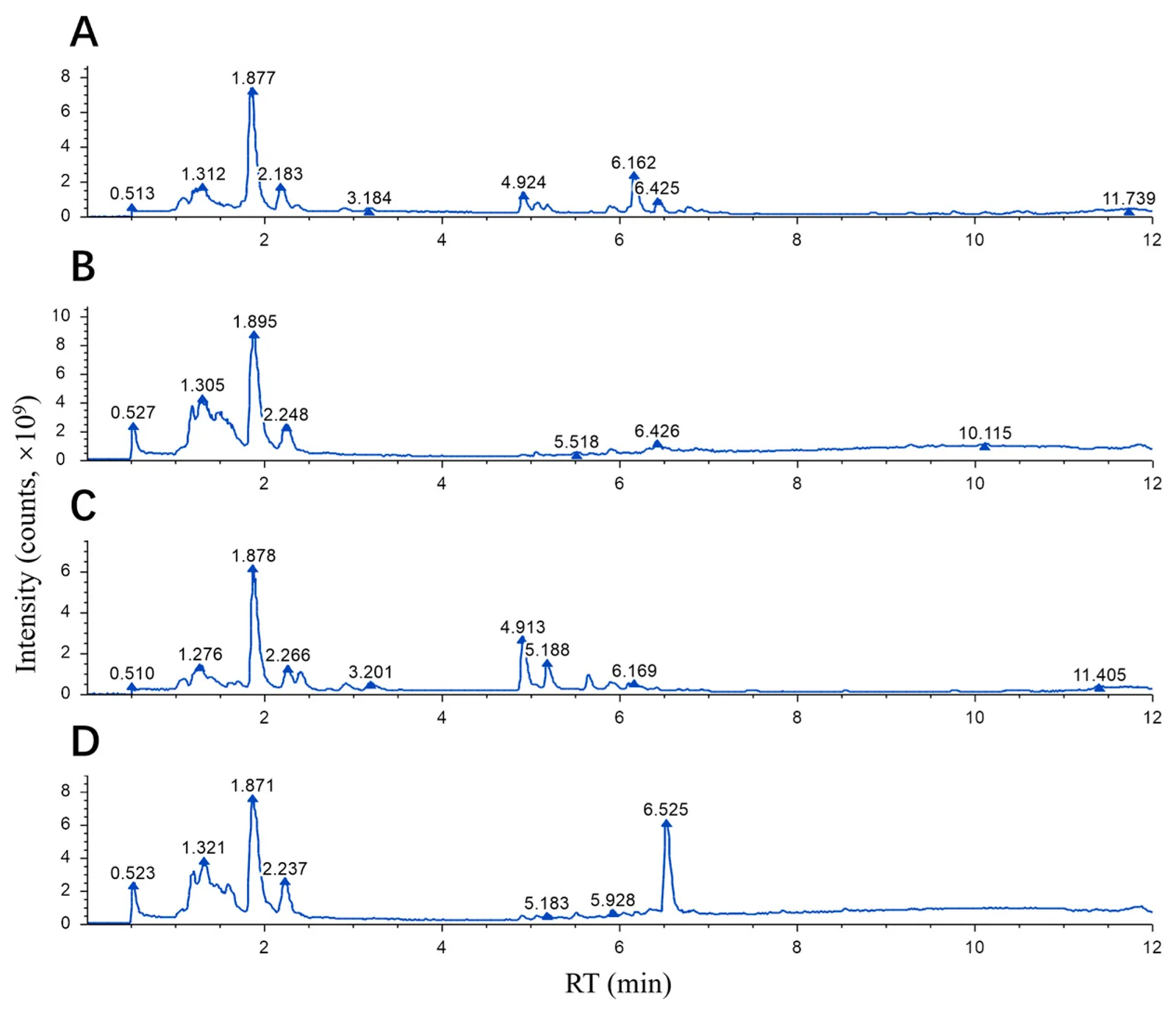
UHPLC-MS/MS ion spectra of metabolites from A431 cells treated with BV ((**A**) positive and (**C**) negative) and solvent ((**B**) positive and (**D**) negative), the arrow tip points to the peak apex, with the time (min) indicated beside it to mark the retention time of this peak.

The total ion chromatograms (TIC) of A431 cells in the BV group and control group under positive and negative electrospray ionization (ESI) modes are shown in Figure 8. The metabolomics detection method has good stability and reproducibility. However, in some retention time intervals, there are significant differences in peak intensity between the two groups of samples, suggesting that BV caused changes in the composition and abundance of metabolites in A431 cells.

Based on the screening criteria of VIP > 1.0, FC > 1.5, or FC < 0.67 and *p* < 0.05, a total of 336 DEMs are identified. The top 15 up-/downregulated DEMs are listed in Table 2. The results of the KEGG pathway enrichment analysis are shown in Table 3. A total of nine significantly enriched metabolic pathways are obtained according to the criterion of Pathway Impact < 0.05, mainly including arginine biosynthesis; phenylalanine, tyrosine and tryptophan biosynthesis; phenylalanine metabolism; arginine and proline metabolism; tryptophan metabolism; glutathione metabolism; purine metabolism; histidine metabolism; and alanine, aspartate and glutamate metabolism.

## 3. Discussion

In our chemical profiling, melittin accounted for 59.84% of the dry weight in the crude *Apis mellifera* venom. While the literature generally reports melittin content in crude bee venom ranging from 40% to more than 63.15%, these values are well-documented under specific biological, environmental, and analytical parameters [29,30]. This higher relative abundance can be attributed to several factors, including the specific geographical origin and subspecies of *Apis mellifera* and seasonal foraging conditions [31]. Melittin is the predominant bioactive peptide responsible for membrane disruption and apoptosis induction [32].

This study found that BV significantly inhibited the proliferation of A431 squamous cell carcinoma cells of the skin in a dose-dependent manner, with an IC_50_ of 11.57 μg/mL. Compared with MCF-7 breast cancer cells, HT-29 colon cancer cells [33], A549 lung cancer cells [34], prostate cancer cells [35], and glioblastoma cells [36] reported in the literature, A431 cells showed higher sensitivity to BV. This differential susceptibility can be rationalized by the unique molecular and membrane characteristics of cSCC. First, A431 cells are characterized by marked epidermal growth factor receptor (EGFR) gene amplification and membrane overexpression [37]. High-density receptor expression in cSCC is closely associated with alterations in cell membrane lipid composition—specifically an enriched presentation of negatively charged phospholipids—which significantly enhances the electrostatic binding and pore-forming efficiency of melittin, the primary active peptide of BV [5,38]. Second, cSCC cells display a pronounced metabolic vulnerability termed arginine dependence, relying heavily on rapid arginine flux for polyamine production and biomass accumulation [39]. Consequently, the targeted depletion of the arginine biosynthetic network induced by BV exerts a disproportionately fatal ‘metabolic starvation’ effect on A431 cells relative to metabolic-flexible cell types [40]. Third, the distinctive background of UV-induced mutations in cSCC renders its signaling architecture highly responsive to BV-triggered TP53 stress networks, thereby lowering the threshold for cell cycle arrest and apoptosis [41,42].

KEGG enrichment analysis using proteomics revealed that DEPs were mainly involved in pathways such as p53 signaling, mineral absorption, and base excision repair. Simultaneously, metabolomics analysis showed that DEMs were mainly enriched in arginine biosynthesis, phenylalanine, tyrosine and tryptophan biosynthesis, and phenylalanine metabolism. The antitumor effect of BV may involve multiple biological processes, including the synergistic regulation of tumor-related signaling pathways, mineral homeostasis, DNA repair-related processes, and amino acid metabolism. This multi-target, multi-pathway regulatory characteristic is consistent with the complex composition and diverse biological activities of BV, providing new evidence for further elucidating its antitumor mechanism.

Among the pathways identified by KEGG proteomics analysis, the p53 signaling pathway was most significantly involved in the antitumor effect of BV on A431 cells. The p53 signaling pathway is an important pathway for regulating cell proliferation and maintaining gene stability. It can participate in various biological processes such as cell cycle arrest, DNA damage repair, cell senescence, and apoptosis by regulating downstream target genes [43]. *TP53* is one of the tumor suppressor genes with the highest mutation frequency in malignant tumors. Its encoded p53 plays an important role in maintaining genome stability [43]. Under stress conditions such as DNA damage, oxidative stress, or oncogene activation, p53 can induce the expression of target genes such as *CDKN1A*, *BAX*, *PUMA*, and *GADD45A*, thereby regulating biological processes such as cell cycle arrest, DNA damage repair, and apoptosis, thus limiting the continuous proliferation of abnormal cells [44,45]. Conversely, *TP53* mutation or *p53* signaling abnormality can weaken the above stress response, promote tumor cell survival, and is closely related to tumor progression and treatment tolerance [46]. In recent years, an increasing number of studies have shown that the function of p53 is not limited to classical cell cycle regulation. It also participates in metabolic processes such as glucose metabolism, lipid metabolism, amino acid metabolism, and redox homeostasis and affects the ability of tumor cells to adapt to nutrient deficiency, oxidative stress, and metabolic stress [47,48]. In addition, p53 has extensive functional connections with signaling pathways such as PI3K/AKT/mTOR, NF-κB, and Hippo, and together they participate in regulating tumor cell proliferation, cell fate determination, and microenvironment adaptation [49]. These studies suggest that the p53 signaling pathway is not only an important regulatory network for cellular stress response but also a key node connecting tumor cell proliferation regulation and metabolic reprogramming.

Beyond its classical role as a master tumor suppressor and orchestrator of cell cycle arrest, TP53 functions as a critical node in cellular metabolic reprogramming under cytotoxic stress. Notably, the p53 signaling pathway emerged as one of the most prominent protein-level cascades, accompanied by alterations in cell cycle and stress response markers such as CCND1 and SERPINE1. Simultaneously, non-targeted UHPLC-MS/MS metabolomics revealed 336 DEMs mapped to key metabolic networks, with arginine biosynthesis (*p* = 1.98 × 10^−5^), phenylalanine, tyrosine and tryptophan biosynthesis (*p* = 3.88 × 10^−4^), and phenylalanine metabolism (*p* = 7.68 × 10^−4^) representing the most profoundly altered pathways.

Importantly, these protein and metabolic networks form a functionally integrated TP53-driven metabolic axis in response to BV. Activated TP53 directly governs nutrient sensing and amino acid flux by transcriptionally regulating metabolic enzymes and transporters, driving cancer cells from an anabolic state toward metabolic crisis [47]. The depletion and structural alteration of arginine, along with aromatic amino acids (phenylalanine, tyrosine, and tryptophan), reflect a p53-mediated restriction of critical building blocks required for rapid A431 cell proliferation and protein synthesis. Furthermore, the downregulation of nuclear CCND1 indicates that p53-driven amino acid starvation functions in synergism with G1/S phase cell cycle arrest [50]. Taken together, our integrated multi-omics analysis demonstrates that BV (rich in melittin) exerts its potent anti-A431 skin cancer effect by engaging a cohesive TP53-centered cascade that directly links protein network signaling to metabolic reprogramming and cell growth inhibition.

Mineral absorption is the second most significant pathway enriched in the proteomics analysis. Although this pathway is mainly related to the transport and homeostasis of essential metal ions, it also includes a variety of proteins involved in redox regulation and cellular stress response [51]. Metallothioneins are cysteine-rich metal-binding proteins that exert antioxidant properties by binding to metals and play an important role in maintaining the homeostasis of metal ions such as zinc and copper, metal detoxification, and resistance to oxidative stress [52]. MT2A is abnormally expressed in a variety of malignant tumors and is closely related to the cell’s ability to adapt to oxidative and metabolic stress [53].

Base excision repair (BER) is another pathway that was significantly enriched in the proteomics analysis. BER is an important repair pathway for DNA base damage in eukaryotic cells, mainly responsible for repairing DNA damage caused by oxidative stress and endogenous metabolism, thereby maintaining genomic stability [54]. The growth and proliferation of tumor cells depend to some extent on the normal function of the DNA repair system, so changes in BER-related proteins may indicate that the DNA repair process is affected [54,55]. The enrichment of the BER pathway in this study suggests that A431 cells may have undergone a DNA repair-related response after BV treatment.

Our subcellular localization analysis of DEPs (Figure 4) provides vital spatial context that strongly aligns with the observed functional phenotypes. Notably, a remarkably high proportion of DEPs were localized to the nucleus. This prominent nuclear enrichment reflects the extensive transcriptional and epigenetic reprogramming triggered by BV treatment in A431 cells. Specifically, the activation of the master transcription factor TP53 leads to its nuclear accumulation, where it directly orchestrates the transcriptional induction or repression of a broad spectrum of nuclear target proteins involved in cell cycle checkpoints (such as CDKN1A/p21) and DNA damage responses [56]. Moreover, the heavy involvement of nuclear proteins corresponds to the suppression of nuclear replication machinery (such as the MCM complex) and chromatin remodeling enzymes, directly driving G1/S phase cell cycle arrest and suppressing DNA synthesis [57]. Thus, the predominant nuclear distribution of DEPs underscores that BV-induced cytotoxicity is orchestrated at the genomic and transcriptional levels within the cell nucleus.

Additionally, the GO enrichment analysis highlighted a prominent cluster related to “transition metal ion binding”, which was accompanied by significant alterations in metallothioneins. Metallothioneins are low-molecular-weight, cysteine-rich proteins essential for maintaining intracellular transition metal homeostasis (particularly for Zn^2+^ and Cu^2+^) and scavenging reactive oxygen species (ROS) [51]. The marked mobilization of MT2A reflects an acute oxidative stress response triggered by BV in A431 cells. Excess or displaced transition metal ions catalyze Fenton-like reactions, leading to intracellular ROS accumulation and severe redox imbalance [58]. While the induction of MT2A serves as an initial antioxidant defense mechanism, sustained BV exposure overrides this buffer capacity, culminating in irreversible oxidative damage, mitochondrial dysfunction, and p53-mediated apoptosis. Thus, the perturbation of transition metal ion binding directly couples metal dyshomeostasis with oxidative stress and apoptotic cell death in BV-treated cSCC cells.

The qRT-PCR analysis revealed that BV exposure led to a marked increase in *CCND1* and *SERPINE1* mRNA levels, matching the corresponding protein changes. In contrast, *TP53* and *IGFBP3* showed no significant mRNA alterations, diverging from their protein expression patterns. Several factors may explain this discrepancy. Post-transcriptional regulation can influence protein abundance; for instance, the copy number of 23–33% of proteins in complexes is subject to such control, despite autosomal gene expression being largely manifested at the protein level [59]. The protein–mRNA correlation is approximately 0.5 [60]; in high-grade serous ovarian cancer, this relationship is weak for structurally stable proteins and mRNA and even weaker for less stable mRNA–protein pairs [61]. Furthermore, reports indicate that in breast cancer, approximately 30% of transcriptional events do not correspond to protein expression [62].

Metabolomics analysis showed that arginine biosynthesis was the most significantly enriched metabolic pathway. Arginine is a semi-essential amino acid that not only participates in protein synthesis but also serves as a precursor for various bioactive molecules such as nitric oxide, polyamines, creatine, and nucleotides. Therefore, it plays an important role in cell proliferation, redox homeostasis, signal transduction, and immune regulation [63]. BV treatment induced a marked depletion of intracellular arginine, accompanied by significant alterations in its key metabolic intermediates, including citrulline and ornithine. Given that ornithine and arginine are rate-limiting substrates for polyamine synthesis (such as putrescine, spermidine, and spermine), their disruption directly impairs polyamine production—a process essential for nucleic acid stabilization and G1/S phase cell cycle transition. Arginine metabolism plays a central role in cellular nitrogen metabolism, and its intermediate products can support multiple biosynthetic pathways. By participating in polyamine synthesis, nucleotide metabolism, and protein biosynthesis, arginine metabolism provides essential metabolic resources for cell biomass accumulation and continuous proliferation [64]. Therefore, arginine metabolism disorder can weaken the metabolic flexibility of tumor cells and limit their ability to adapt to the stress microenvironment [65,66]. This study found that arginine-related metabolites changed significantly after BV treatment, suggesting that BV affects arginine utilization and biosynthetic metabolic processes in A431 cells. Beyond arginine metabolism, BV treatment triggered targeted alterations across the broader amino acid network, particularly within the aromatic amino acid pathways.

The second most significantly enriched metabolic pathway is phenylalanine, tyrosine, and tryptophan biosynthesis. Aromatic amino acids play important roles in cellular metabolic regulation, redox homeostasis, and stress response, in addition to their role in protein synthesis [67,68]. The identification of TP53 alterations is closely related to tumor metabolic reprogramming. Among them, tryptophan metabolism is closely related to tumor progression and immune regulation [69], while phenylalanine and tyrosine metabolism are involved in maintaining cellular metabolic homeostasis [70]. The tryptophan-kynurenine axis, driven by indoleamine 2,3-dioxygenase 1, serves as a crucial metabolic checkpoint regulating cellular redox balance and stress adaptation [71]. Coupled with marked fluctuations in phenylalanine and tyrosine levels that impair general translation machinery, these specific metabolite shifts demonstrate that BV exerts its potent anti-A431 activity by perturbing overall amino acid homeostasis and starving cells of critical proliferative precursors. In addition, aromatic amino acid metabolism is closely related to multiple metabolic processes such as redox regulation, so changes in this pathway may also participate in the overall metabolic response induced by BV [68].

Crucially, these specific metabolite fluctuations directly converge with our proteomic findings, underscoring a functional cross-talk between p53 transcriptional signaling and amino acid metabolic reprogramming. The activation of TP53 serves as a pivotal metabolic master switch in BV-treated A431 cells. By regulating downstream metabolic enzymes involved in the urea cycle and amino acid processing, p53-driven transcriptional control actively suppresses arginine biosynthesis and restricts nitrogen flux required for polyamine and protein synthesis. Simultaneously, p53 activation impacts tryptophan catabolism via the IDO1/kynurenine axis, depriving A431 cells of essential survival metabolites and inducing metabolic crisis. Together, these integrated multi-omics results demonstrate that BV exerts its antitumor efficacy by triggering a tightly coordinated TP53-centered cascade, which bridges protein signaling networks directly with amino acid starvation and growth inhibition.

This study still has certain limitations. First, a key limitation of this study is the reliance on a single IC_50_ concentration and a fixed 24 h time point for omics analyses, which makes it challenging to separate early upstream drivers from secondary cellular stress responses. Additionally, further functional validations, such as gene knockout or metabolic enzyme inhibition, are warranted to confirm pathway dependencies. Future work incorporating dynamic time-series sampling and multi-dose gradients will help map the precise kinetics of BV action. Second, this study only evaluated the antitumor effect of BV against A431 cells. Future research could explore the efficacy of combining BV with existing treatments across a broader panel of cSCC cell lines to assess its synergistic antitumor effect. Finally, the involvement of the TP53 stress network and base excision repair cascades was identified primarily through high-throughput label-free proteomics and untargeted metabolomics profiling. While quantitative mass spectrometry provided robust peptide-level evidence for key node alterations, future studies utilizing targeted assays—such as Western blotting and genetic knockdown/knockout models for p53 and p21—will be necessary to further delineate the exact molecular hierarchy governing BV-induced cell cycle arrest and apoptotic death in cSCC.

## 4. Materials and Methods

### 4.1. Collection of BV and Detection of Melittin Content

The thorax and abdomen of adult worker bees (*Apis mellifera*) were fixed with tweezers, and the tail was stimulated. Each individual bee was squeezed until a droplet could be collected from the tip of the sting into a culture dish. The dish was then dried in a fume hood and stored at −20 °C.

The content of melittin in bee venom was determined by high-performance liquid chromatography (HPLC) [72]. The chromatographic column was XB-C18 (4.6 mm × 250 mm), the mobile phase A was 0.1% (*v*/*v*) trifluoroacetic acid (TFA) aqueous solution, and the mobile phase B was acetonitrile solution containing 0.1% (*v*/*v*) trifluoroacetic acid. Both were filtered through a 0.22 μm filter membrane (Biosharp Beijing Labgic Technology Co., Ltd., Beijing, China) before use. The flow rate was set to 1.0 mL/min, the column temperature was maintained at 30 °C, the detection wavelength was 220 nm, and the injection volume was 5 μL. The gradient elution program was as follows: 0–7 min, phase B linearly increased from 20% to 70%; 7–8 min, decreased from 70% to 20%; 8–15 min, maintained at 20% B to equilibrate the column. Melittin standard (Shanghai Aladdin Biochemical Technology Co., Ltd., Shanghai, China) was prepared into standard solutions of 20, 50, 100, 200, 500, and 2000 μg/mL, respectively, and detected under the above chromatographic conditions. A standard curve was established with the concentration of melittin as the *x*-axis and peak area as the *y*-axis. BV samples were dissolved in ultrapure water and prepared to a concentration of 3 mg/mL. After HPLC detection, the actual concentration of melittin in the sample was calculated by substituting the measured peak area into the standard curve. The melittin content was calculated using the standard curve equation.

### 4.2. Antitumor Bioassay

The antitumor activity of BV against A431 cells (purchased from Wuhan Purosai Life Sciences Co., Ltd., Wuhan, China) was determined according to the method previously reported by our research group, with appropriate adjustments [73]. BV was prepared into a 1000 μg/mL stock solution using an A431 cell-specific culture medium (Procell Life Science & Technology Co., Ltd., Wuhan, China). The solution was then sonicated at 40 kHz for 30 min (KQ-500DE, Kunshan Ultrasonic Instruments Co., Ltd., Kunshan, China). After filtration through a 0.22 μm filter membrane, aliquots were prepared and stored at −20 °C for further experiments. Before the experiment, the stock solution was diluted to the required concentration with the specific culture medium.

A431 cells were washed 2–3 times with PBS buffer (phosphate-buffered saline, PBS, pH 7.2–7.4, Sinopharm Chemical Reagent Co., Ltd., Shanghai, China) and then digested with 0.25% trypsin (HyClone, Thermo Scientific, Waltham, MA, USA). The cells were resuspended in complete specific culture medium and centrifuged at 137× *g* for 5 min (TDZ4-WS, Hunan Xiang Yi Laboratory Instrument Development Co., Ltd., Changsha, China). Cell density was determined via 0.4% trypan blue (Beijing Solarbio Science & Technology Co., Ltd., Beijing, China) and adjusted to 5 × 10^4^ cells/mL. Each well of a 96-well plate was seeded with 100 μL cell suspension and cultured at 37 °C in a 5% CO_2_ incubator (C150, BINDER GmbH, Tuttlingen, Baden-Württemberg, Germany) for 24 h.

BV stock solution was diluted to 8, 10, 12, 14, and 16 μg/mL using the specific culture medium. These BV solutions (100 μL) and the specific culture medium (100 μL) were separately added to each well to form treatment groups and a blank control group. The morphology of cells was observed and recorded using an inverted microscope (TS-100f, NIKON CORPORATION, Tokyo, Tokyo, Japan) after 24 h of cell culture. These cells were washed with PBS after the removal of culture medium, and then 110 μL of a complete culture medium containing 10 μL of CCK-8 reagent (Dojindo Laboratories, Kumamoto, Kumamoto, Japan) was added to each well. After incubation in the dark for 2 h, the absorbance (OD) was measured at 450 nm using a microplate reader (1510, Thermo Fisher Scientific, Waltham, MA, USA). The cell proliferation inhibition rate was calculated using the following formula: Cell proliferation inhibition rate (%) = [(Blank control group OD − Treatment group OD)/(Blank control group OD − Blank group OD)] × 100%. The IC_50_ of BV on A431 cells was calculated based on the cell viability after treatment with different concentrations of BV. Six replicates were set up for each group.

### 4.3. Differentially Expressed Proteins in A431 Cells Treated with BV

A431 cells were treated according to the morphology experimental methods with the blank control group and an IC_50_ (11.57 µg/mL) BV group. The BV remained in the culture system throughout the 24 h treatment. After 24 h of cell culture, the cells were washed twice with pre-cooled PBS buffer after the culture medium was discarded, followed by trypsin digestion (0.25% trypsin for 5 min) and washing again. After centrifugation at 137× *g* for 5 min, the cell pellet was collected and immediately frozen in liquid nitrogen and stored at −80 °C for later use. Their total proteins were extracted according to a previously described method [74]. The concentration of total proteins was determined by the Coomassie Brilliant Blue method. The spectra of proteins in A431 cells were determined using LC-MS-MS with a Q Exactive HF-X mass spectrometer (Thermo Fisher, Waltham, MA, USA) with a Nanospray Flex™ electrospray ionization (ESI) source. The spray voltage was set to 2.1 kV, and the ion transfer tube temperature was set to 320 °C. Data-dependent acquisition (DDA) was used. The full MS scan range was *m*/*z* 350–1500, the resolution was set to 60,000 (at *m*/*z* 200), the AGC target was 3 × 10^6^, and the maximum ion injection time was 20 ms. In each full-scan cycle, the top 40 precursor ions with the highest signal intensities were selected for higher-energy collisional dissociation (HCD). The MS/MS resolution was set to 15,000 (at *m*/*z* 200), the AGC target was 1 × 10^5^, the maximum ion injection time was 45 ms, the normalized collision energy was set to 27%, the intensity threshold was 2.2 × 10^4^, and the dynamic exclusion time was set to 20 s. The resulting raw mass spectrometry data were saved in .raw format.

### 4.4. Differentially Expressed Metabolites in A431 Cells Treated with BV

The determination of DEMs followed the previous study [75]. Similar to the proteomics protocol, A431 cells cultured in the BV group (IC_50_ 11.57 µg/mL) and blank control group were collected and resuspended in 300 µL of pre-cooled 80% methanol (Thermo Fisher Scientific, Waltham, MA, USA). The samples were snap-frozen in liquid nitrogen for 5 min, thawed on ice, vortexed for 30 s, and subjected to ultrasonication for 6 min. Following sonication, the cell lysates were centrifuged at 5971× *g* for 1 min at 4 °C using a D3024R refrigerated centrifuge (Scilogex, Rocky Hill, CT, USA). The resulting supernatants were collected and lyophilized into dry powder. All dried samples were reconstituted in a volume of 10% methanol equivalent to 10% of their original sample volume prior to LC-MS analysis. Quality control (QC) samples were prepared by pooling equal-volume aliquots from each experimental sample to monitor system stability.

Liquid chromatography–tandem mass spectrometry (LC-MS/MS) analysis was performed using a Vanquish UHPLC system coupled to a Q Exactive™ HF-X Mass Spectrometer (Thermo Fisher Scientific, USA). Chromatographic separation was achieved on a Hypersil Gold column (100 × 2.1 mm, 1.9 µm particle size, Thermo Fisher Scientific) maintained at 40 °C with a constant flow rate of 0.2 mL/min. In positive electrospray ionization (ESI+) mode, mobile phase A consisted of 0.1% formic acid in water, and mobile phase B was methanol. In ESI− mode, mobile phase A consisted of 5 mM ammonium acetate (pH 9.0), and mobile phase B was methanol. The chromatographic gradient elution program was set as follows: 0.0–1.5 min, 98% A and 2% B; 1.5–3.0 min, linear gradient to 0% A and 100% B; 3.0–10.0 min, maintained at 0% A and 100% B; 10.0–10.1 min, returned to 98% A and 2% B; 10.1–12.0 min, re-equilibration with 98% A and 2% B.

Mass spectrometry was operated in data-dependent acquisition (DDA) mode across a scan range of *m*/*z* 100–1500 for both full MS and MS/MS secondary scans. The ESI source parameters were optimized as follows: spray voltage of 3.5 kV, sheath gas flow rate of 35 psi, auxiliary gas flow rate of 10 L/min, capillary temperature of 320 °C, S-lens RF level of 60, and auxiliary gas heater temperature of 350 °C. Raw data files (.raw) were imported into Compound Discoverer 3.1 (Thermo Scientific) for metabolite identification and relative quantification. Subsequent statistical and bioinformatic data processing was executed on a Linux operating system (CentOS version 6.6) using R (v3.4.3) and Python (v2.7.6) environment scripts.

#### Expression of the Hub Genes

To validate the representative differentially expressed proteins screened by label-free proteomics, the qRT-PCR experiment used the same cell treatment conditions as the proteomics experiment. The primer sequences were designed using the Primer-BLAST (Primer3 v1.1.0, BLAST+ v2.16.0; Primer-BLAST, National Center for Biotechnology Information, Bethesda, MD, USA) tool from the National Center for Biotechnology Information (NCBI) [76]. With TBP as the reference gene, the relative mRNA expression levels of CCND1, SERPINE1, TP53, and IGFBP3 were calculated [77]. The primers (Table 4) were synthesized by Fuzhou Shangya Biotechnology Co., Ltd. (Fuzhou, China). Three biological replicates were set up for each group.

The experimental methods followed the MIQE 2.0 guidelines [78]. Total RNA was extracted from A431 cells using a total RNA extraction kit (Promega (Beijing, China) Biotech Co., Ltd., Beijing, China), and RNA concentration and purity were measured using a microvolume UV spectrophotometer (NanoDrop One, Thermo Fisher Scientific, Waltham, MA, USA). Reverse transcription was performed using the HiFair^®^ Advance Fast 1st Strand cDNA Synthesis Kit (Yeasen Biotechnology (Shanghai) Co., Ltd., Shanghai, China) in a PCR instrument (2720 Thermal Cycler, Applied Biosystems, Carlsbad, CA, USA). Subsequently, qRT-PCR detection was carried out using Hieff^®^ UNICON^®^ Universal Blue qPCR SYBR Green Master Mix (Yeasen Biotechnology (Shanghai) Co., Ltd., Shanghai, China) on a real-time quantitative PCR system (CFX384™ Optics Module, Bio-Rad Laboratories, Inc., Hercules, CA, USA).

### 4.5. Data Analysis

All experiments were performed at least in triplicate independently. Results are expressed as mean ± standard error (Mean ± SEM). One-way ANOVA and the IC_50_ of BV in A431 cells were performed using GraphPad Prism 8.0.2 (GraphPad Software, Inc., San Diego, CA, USA).

Proteomics data were analyzed using Proteome Discoverer 2.2 software (Thermo Fisher Scientific, Bremen, Germany) in the homo_sapiens_uniprot_2022_1_27 database for protein identification and quantification. Search parameters were set as follows: precursor ion mass error 10 ppm, fragment ion mass error 0.02 Da, fixed modification as cysteine alkylation, variable modifications including methionine oxidation and N-terminal acetylation, and a maximum of two missed cleavage sites allowed. Further quality control was performed using the False Discovery Rate (FDR), retaining only proteins with a confidence level greater than 99% and containing at least one specific peptide for subsequent analysis. A t-test was used to screen DEPs. To further ensure statistical robustness and eliminate potential single-protein false-positive annotations, DEPs only enriched by a single protein as well as non-relevant organ-specific disease terms were removed.

Metabolomics data were annotated using the KEGG, HMDB, and LIPID MAPS databases. Principal component analysis (PCA) and partial least squares discriminant analysis (PLS-DA) were performed using MetaX software (v2.0), combined with variable projection importance (VIP), fold change (FC), and statistical significance to screen DEMs. Screening criteria were set as VIP > 1.0, FC > 1.5, or FC < 0.67, with *p* < 0.05. Pathway enrichment analysis of differentially expressed metabolites was performed using the MetaboAnalyst platform (https://www.metaboanalyst.ca/, accessed on 15 April 2022), identifying significantly affected metabolic pathways through pathway enrichment and topological analysis.

## 5. Conclusions

This study demonstrates that BV significantly inhibits the growth of A431 cells in vitro with an IC_50_ of 11.57 μg/mL. Combined label-free proteomics and untargeted metabolomics analyses revealed that BV-induced cytotoxicity is potentially driven by a cohesive TP53-centered stress response that directly orchestrates metabolic reprogramming. Specifically, BV treatment activates the p53 signaling network and causes targeted exhaustion of the arginine biosynthetic pathway (disrupting citrulline, ornithine, and downstream polyamine supply) alongside shifts in the tryptophan/kynurenine axis. But the transcriptomic data failed to confirm *TP53* upregulation. These findings provide a valuable reference for apipuncture and the further development of BV-derived antitumor drugs.

## Figures and Tables

**Table 1:** The top 15 up-/downregulated DEPs of A431 cells from the BV group compared with the blank control group.

UniProt ID	Gene Symbol	Name of Protein	Fold Change	*p*	Up-/Downregulated
B4E295	—	Calcium-transporting ATPase	Inf	0	up
A0A024R702	*CGI-38*	Tubulin polymerization-promoting protein family member 3	Inf	0	up
P53609	*PGGT1B*	Geranylgeranyl transferase type-1 subunit beta	Inf	0	up
B3KPL2	*PHF15*	PHD finger protein 15, isoform CRA_a	Inf	0	up
O60583	*CCNT2*	Cyclin-T2	Inf	0	up
Q9NX20	*MRPL16*	39S ribosomal protein L16, mitochondrial	1.65	1.48 × 10^−4^	up
Q6FI00	*CCND1*	CCND1 protein	2.91	2.08 × 10^−4^	up
Q7KZ85	*SUPT6H*	Transcription elongation factor SPT6	2.09	4.07 × 10^−4^	up
A0A024QYT5	*SERPINE1*	Serpin peptidase inhibitor, clade E (Nexin, plasminogen activator inhibitor type 1), member 1, isoform CRA_b	4.00	4.59 × 10^−4^	up
Q15067	*ACOX1*	Peroxisomal acyl-coenzyme A oxidase 1	1.84	8.62 × 10^−4^	up
B3KQ21	—	cDNA FLJ32640 fis, clone SYNOV2001033, highly similar to U3 small nucleolar RNA-associated protein 6 homolog	1.93	9.76 × 10^−4^	up
B2RAZ5	*B4GALT4*	UDP-Gal:betaGlcNAc beta 1,4-galactosyltransferase, polypeptide 4	2.30	1.25 × 10^−3^	up
Q92544	*TM9SF4*	Transmembrane 9 superfamily member 4	1.54	2.35 × 10^−3^	up
Q96L58	*B3GALT6*	Beta-1,3-galactosyltransferase 6	2.36	3.07 × 10^−3^	up
A0A024R219	*ZA20D2*	Zinc finger, A20 domain containing 2, isoform CRA_a	1.90	3.16 × 10^−3^	up
A0A7I2YQ88	*PABPC1*	Polyadenylate-binding protein	Inf	0	down
Q13153	*PAK1*	Serine/threonine-protein kinase PAK 1	Inf	0	down
A0A024R8Y2	*POU2F1*	POU domain protein	Inf	0	down
O14641	*DVL2*	Segment polarity protein dishevelled homolog DVL-2	Inf	0	down
Q15276	*RABEP1*	Rab GTPase-binding effector protein 1	Inf	0	down
A0A024R3L9	*VPS26B*	Vacuolar protein sorting 26 homolog B (Yeast), isoform CRA_a	Inf	0	down
O00418	*EEF2K*	Eukaryotic elongation factor 2 kinase	Inf	0	down
P78310	*CXADR*	Coxsackievirus and adenovirus receptor	Inf	0	down
Q9BQC6	*MRPL57*	Ribosomal protein 63, mitochondrial	Inf	0	down
Q5T5X7	*BEND3*	BEN domain-containing protein 3	Inf	0	down
D3VVP5	*ATXN3*	Ubiquitinyl hydrolase 1 (Fragment)	0.52	2.96 × 10^−4^	down
Q8NI60	*COQ8A*	Atypical kinase COQ8A, mitochondrial	0.66	2.97 × 10^−4^	down
Q5JSZ5	*PRRC2B*	Protein PRRC2B	0.46	3.65 × 10^−4^	down
A0A1B0GVH5	*FTO*	Alpha-ketoglutarate-dependent dioxygenase FTO	0.35	4.65 × 10^−4^	down
B4E218	—	cDNA FLJ61052, highly similar to Homo sapiens myosin head domain containing 1 (MYOHD1), transcript variant 2, mRNA	0.54	5.66 × 10^−4^	down

Note: “—” means no Gene Symbol of the protein.

**Table 2:** The top 15 up-/downregulated DEMs of A431 cells from the BV group compared with the blank control group.

Compound Name	HMDB_ID	Fold Change	*p*	VIP	Ion Mode
(2S)-4-Oxo-2-phenyl-3,4-dihydro-2H-chromen-7-yl beta-D-glucopyranoside	—	24.93	5.40 × 10^−9^	1.69	neg
D-Raffinose	HMDB0003213	3.11	6.91 × 10^−8^	1.43	pos
11-Dehydro thromboxane B2	—	6.11	2.21 × 10^−7^	1.67	neg
Maltotetraose	HMDB0001296	2.99	3.56 × 10^−7^	1.41	pos
2-{[(4,5-dimethoxy-2-nitrophenethyl)imino]methyl}phenol	—	16.03	4.29 × 10^−7^	1.42	pos
C-8 Ceramide-1-phosphate	—	8.73	4.79 × 10^−7^	1.41	pos
YQH	—	8.56	5.84 × 10^−7^	1.41	pos
Carminic acid	HMDB0030658	14.95	6.42 × 10^−7^	1.67	neg
Arachidonic acid	HMDB0001043	23.80	1.07 × 10^−6^	1.64	neg
Glycohyocholic acid Sodium salt	—	15.46	1.18 × 10^−6^	1.41	pos
Cinnamoylglycine	HMDB0011621	2.83	2.56 × 10^−6^	1.42	pos
PC (18:0e/2:0)	—	4.49	3.02 × 10^−6^	1.39	pos
Υ-L-Glutamyl-L-glutamic acid	—	2.31	3.62 × 10^−6^	1.38	pos
Isorhamnetin	HMDB0002655	8.16	5.85 × 10^−6^	1.65	neg
D-(+)-Maltose	HMDB0000163	2.54	5.88 × 10^−6^	1.38	pos
beta-Nicotinamide mononucleotide	HMDB0000229	0.15	8.77 × 10^−9^	1.44	pos
N2-tetrahydrofuran-2-ylmethyl-4-(4-fluorophenyl)-1,3-thiazol-2-amine	—	0.07	2.03 × 10^−8^	1.43	pos
Pro-Leu	HMDB0253028	0.08	2.24 × 10^−8^	1.44	pos
2-(1-benzofuran-2-yl)-4H-chromen-4-one	—	0.04	3.81 × 10^−8^	1.44	pos
16-Heptadecyne-1,2,4-triol	—	0.03	5.70 × 10^−8^	1.45	pos
2-Hydroxycaproic acid	HMDB0001624	0.40	6.01 × 10^−8^	1.66	neg
Ecgonine	HMDB0006548	0.04	6.71 × 10^−8^	1.45	pos
Monolaurin	HMDB0245396	0.01	2.06 × 10^−7^	1.45	pos
Methyl dihydrojasmonate	HMDB0031740	0.03	2.97 × 10^−7^	1.44	pos
7-(1-pyrrolidinyl)pyrimido[4,5-d]pyrimidin-4-amine	—	0.03	7.27 × 10^−7^	1.41	pos
L-Histidine	HMDB0000177	0.15	8.60 × 10^−7^	1.40	pos
Dl-Lanthionine	HMDB0251521	0.05	1.03 × 10^−6^	1.45	pos
2,4-Dichlorophenol	HMDB0004811	0.02	1.09 × 10^−6^	1.64	neg
Isoproterenol	HMDB0015197	0.12	1.81 × 10^−6^	1.42	pos
tetranor-12(R)-HETE	—	0.02	2.81 × 10^−6^	1.41	pos

Note: “—” means no HMDB_ID of the DEM.

**Table 3:** Significantly enriched pathways of DEMs.

Pathway Name	*p* Value
Arginine biosynthesis	0.000019796
Phenylalanine, tyrosine and tryptophan biosynthesis	0.00038799
Phenylalanine metabolism	0.00076753
Arginine and proline metabolism	0.0015008
Tryptophan metabolism	0.0023856
Glutathione metabolism	0.0084879
Purine metabolism	0.02981
Histidine metabolism	0.036083
Alanine, aspartate and glutamate metabolism	0.039322

**Table 4:** Primer sequences used for qRT-PCR.

Gene	Forward Primer (5′-3′)	Reverse Primer (5′-3′)
*TBP*	ACTCCACTGTATCCCTCCCC	TATATTCGGCGTTTCGGGCA
*CCND1*	GAGGCGGAGGAGAACAAACA	GGAGGGCGGATTGGAAATGA
*SERPINE1*	AAAGGCAACATGACCAGGCT	TCTAGGGCTTCCTGAGGTC
*TP53*	TGAAGCTCCCAGAATGCCAG	GCTGCCCTGGTAGGTTTTCT
*IGFBP3*	AGAAAAAGCAGTGTCGCCCT	CTTCCCCTTGGTGGTGTAGC

## Data Availability

Data are contained within this article.

## Supplementary Materials

The following supporting information can be downloaded at: https://www.mdpi.com/article/10.3390/ijms27198725/s1.

## Abbreviations

The following abbreviations are used in this manuscript:

cSCC	Cutaneous squamous cell carcinoma
BV	Bee venom
DEPs	Differentially expressed proteins
DEMs	Differentially expressed metabolites
BER	Base excision repair
