# Supplementary material for: Inhibitory Mechanism of Bee Venom Against A431 Cutaneous Squamous Cell Carcinoma Cells Based on Label-Free Proteomics and Untargeted Metabolomics
Source: Int J Mol Sci. 2026 Sep 29;27(19):8725. doi: 10.3390/ijms27198725 (PMC13665046; doi:10.3390/ijms27198725)
Supplement: Supplementary file 1 [file ijms-27-08725-s001.zip › ijms-4484265-supplementary.pdf]

Article

Inhibitory Mechanism of Bee Venom Against A431 Cutaneous Squamous Cell Carcinoma Cells Based on Label-Free Proteomics and Untargeted Metabolomics

Ao Yang †, Jingjing Li † and Wenchao Yang *

College of Bee Science, Fujian Agriculture and Forestry University, Fuzhou 350002, China; ya104110@126.com (A.Y.); lijingjing000407@163.com (J.L.)

* Correspondence: beesyang@gmail.com

† These authors contributed equally to this work.

Table S1. The significantly enriched pathways ($p < 0.05$) of differentially expressed proteins.

No	MapTitle	p	Description
1	p53 signaling pathway	1.72×10^{-3}	Serpin peptidase inhibitor, clade E (Nexin, plasminogen activator inhibitor type 1), member 1, isoform CRA_b
			Cellular tumor antigen p53
			CCND1 protein
			Insulin-like growth factor-binding protein 3
2	Mineral absorption	2.89×10^{-3}	Metallothionein-2
			Heme oxygenase
			Metallothionein-1F
3	Base excision repair	4.31×10^{-3}	DNA ligase 1
			DNA polymerase
			Protein mono-ADP-ribosyltransferase PARP4
4	Mitophagy - animal	6.74×10^{-3}	Tax1-binding protein 1
			Cellular tumor antigen p53
			Gamma-aminobutyric acid receptor-associated protein-like 2
			cDNA FLJ75601, highly similar to Homo sapiens neighbor of BRCA1 gene 1 (NBR1), transcript variant 1, mRNA
5	Transcriptional misregulation in cancer	1.26×10^{-2}	Cellular tumor antigen p53
			Histone H3.1
			Insulin-like growth factor-binding protein 3
			Cyclin-T2
6	Basal cell carcinoma	1.48×10^{-2}	Cellular tumor antigen p53

			Segment polarity protein dishevelled homolog DVL-2<br>Serpin peptidase inhibitor, clade E (Nexin, plasminogen activator inhibitor type 1), member 1, isoform CRA_b
7	Cellular senescence	1.88×10^{-2}	Cellular tumor antigen p53<br>CCND1 protein<br>Insulin-like growth factor-binding protein 3<br>Cyclin-A2
8	Glycosphingolipid biosynthesis - lacto and neolacto series	2.15×10^{-2}	UDP-Gal:betaGlcNAc beta 1,4-galactosyltransferase, polypeptide 4
9	One carbon pool by folate	2.20×10^{-2}	Thymidylate synthase<br>Serine hydroxymethyltransferase, cytosolic
10	Retinol metabolism	2.20×10^{-2}	Cytochrome P450 2S1<br>Short-chain dehydrogenase/reductase 3
11	Antifolate resistance	3.98×10^{-2}	Thymidylate synthase<br>Serine hydroxymethyltransferase, cytosolic
12	MicroRNAs in cancer	4.24×10^{-2}	Heme oxygenase<br>Cellular tumor antigen p53<br>CCND1 protein<br>Programmed cell death protein 4
13	Mismatch repair	4.49×10^{-2}	DNA ligase 1<br>DNA polymerase
14	Complement and coagulation cascades	4.49×10^{-2}	Serpin peptidase inhibitor, clade E (Nexin, plasminogen activator inhibitor type 1), member 1, isoform CRA_b<br>Complement decay-accelerating factor
15	Proteoglycans in cancer	4.72×10^{-2}	Serine/threonine-protein kinase PAK 1<br>Cellular tumor antigen p53<br>CCND1 protein<br>Diphtheria toxin receptor (Heparin-binding epidermal growth factor-like growth factor) variant (Fragment)<br>Programmed cell death protein 4
